# Proximal Policy Optimization-based Task Offloading Framework for Smart Disaster Monitoring using UAV-assisted WSNs

**DOI:** 10.1016/j.mex.2025.103472

**Published:** 2025-06-26

**Authors:** C.N. Vanitha, P. Anusuya, Rajesh Kumar Dhanaraj, Dragan Pamucar, Mahmoud Ahmad Al-Khasawneh

**Affiliations:** a Department of Information Technology, Karpagam College of Engineering, Coimbatore, India; b Symbiosis Institute of Computer Studies and Research (SICSR), Symbiosis International (Deemed University), Pune, India; c Szechenyi Istvan University, Gyor, Hungary; d School of Computing, Skyline University College, University City Sharjah, 1797 Sharjah, United Arab Emirates; e Applied Science Research Center. Applied Science Private University, Amman, Jordan

**Keywords:** UAV assisted WSNs, Task offloading, Edge computing, Reinforcement learning, Disaster monitoring, Proximal policy optimization (PPO), ETORL-UAV integrating PPO

## Abstract

Unmanned Aerial Vehicles (UAVs) are increasingly employed in Wireless Sensor Networks (WSNs) to enhance communication, coverage, and energy efficiency, particularly in disaster monitoring and remote surveillance scenarios. However, challenges such as limited energy resources, dynamic task allocation, and UAV trajectory optimization remain critical. This paper presents Energy-efficient Task Offloading using Reinforcement Learning for UAV-assisted WSNs (ETORL-UAV), a novel framework that integrates Proximal Policy Optimization (PPO) based reinforcement learning to intelligently manage UAV-assisted operations in edge-enabled WSNs. The proposed approach utilizes a multi-objective reward model to adaptively balance energy consumption, task success rate, and network lifetime. Extensive simulation results demonstrate that ETORL-UAV outperforms five state-of-the-art methods Meta-RL, g-MAPPO, Backscatter Optimization, Hierarchical Optimization, and Game Theory based Pricing achieving up to 9.3 % higher task offloading success, 18.75 % improvement in network lifetime, and 27 % reduction in energy consumption. These results validate the framework's scalability, reliability, and practical applicability for real-world disaster-response WSN deployments.

• Proposes ETORL-UAV: Energy-efficient Task Offloading using Reinforcement Learning for UAV-assisted WSNs
• Leverages PPO-based reinforcement learning and a multi-objective reward model
• Demonstrates superior performance over five benchmark approaches in disaster-response simulations

## Graphical abstract

**Figure:**
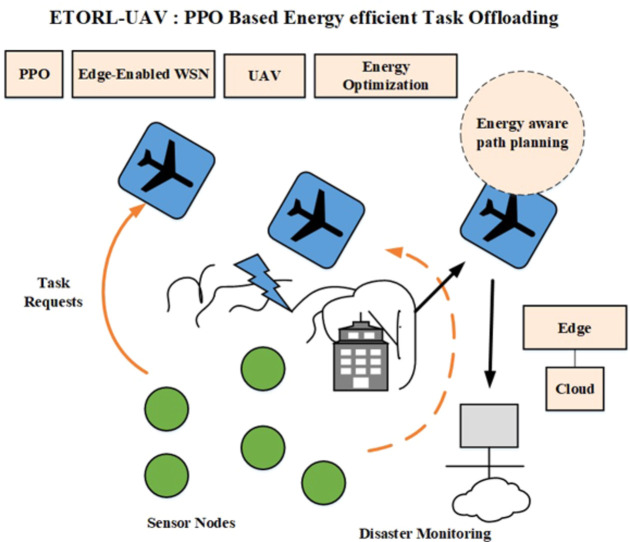

Specifications table

**Table:** 

Subject area:	Engineering
More specific subject area:	Wireless Sensor Networks, Artificial Intelligence, Reinforcement Learning, Edge Computing, Disaster Monitoring,
Name of your method:	ETORL-UAV integrating PPO
Name and reference of original method:	L. Zhang *et al*., "Task Offloading and Trajectory Control for UAV-Assisted Mobile Edge Computing Using Deep Reinforcement Learning," in *IEEE Access*, vol. 9, pp. 53,708–53,719, 2021, doi:10.1109/ACCESS.2021.3070908.
Resource availability:	The ETORL-UAV framework is developed and tested using Python-based simulations with Gym and Stable-Baselines3 libraries. It utilizes Proximal Policy Optimization (PPO) for training UAV agents and models an edge-enabled UAV-WSN environment with energy and mobility constraints. The simulation setup ensures reliable evaluation under diverse disaster-response scenarios. PPO implementation via Stable-Baselines3 Simulation using Python, Gym

## Background

**Fig. 1:**
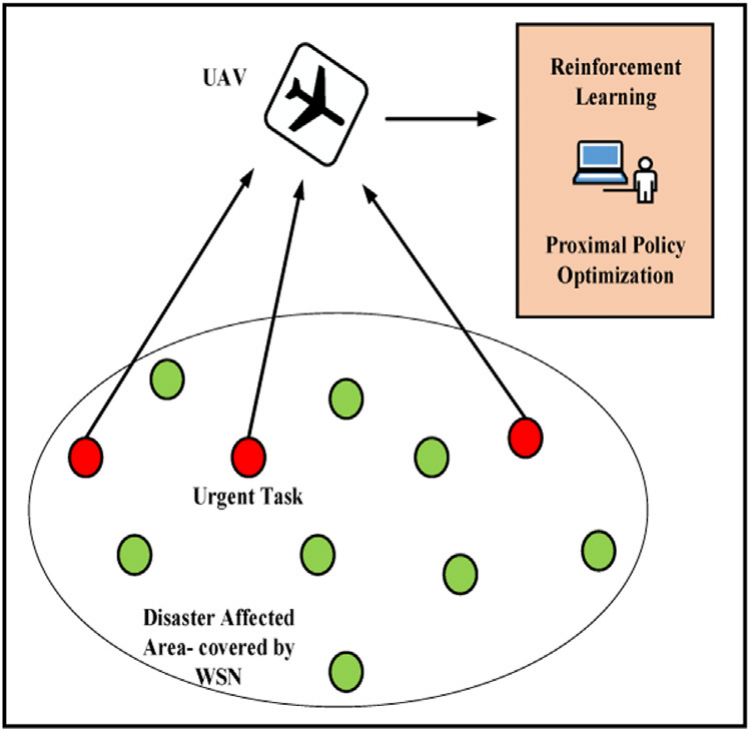
Network Scenario.

WSNs have become integral to various real-time monitoring applications, including environmental surveillance, industrial automation, agriculture, and disaster response [1,2]. In the context of disaster monitoring such as earthquake detection, flood alerts, wildfire tracking, or structural failure warnings, WSNs offer timely and critical situational data to support decision-makers and first responders [3,4]. These networks consist of spatially distributed sensor nodes that collaboratively sense, process, and transmit environmental data as shown in Fig. 1. However, sensor nodes are inherently limited in terms of energy capacity, computational resources, memory, and communication range [5,6]. These limitations are exacerbated in dynamic and harsh disaster environments, where infrastructure may be damaged, energy replenishment is infeasible, and communication channels are unreliable [7].

To mitigate these constraints, the Mobile Edge Computing (MEC) enables data processing at the network edge closer to the data source thereby reducing reliance on distant cloud servers, minimizing latency, and improving Quality of Service (QoS). In disaster settings, deploying traditional static edge servers is not feasible due to the unpredictability and inaccessibility of affected areas. This has led to the emergence of UAVs as mobile edge computing platforms [8]. UAVs offer the advantages of mobility, flexibility, line-of-sight communication, and on-board processing capabilities, making them ideal for operating in disaster-affected regions [9].

In a UAV-assisted edge-enabled WSN, UAVs can hover over or move around the disaster zone, collect data from sensor nodes, and process tasks on-board or relay them to more powerful computing infrastructures if necessary [10]. Despite these advantages, the major challenges in such a setup is task offloading the decision-making process of whether a computational task should be processed locally at the sensor node, offloaded to a UAV. Task offloading is influenced by various factors including network congestion, node energy levels, UAV availability, link quality, task urgency, and processing requirements.

Traditional task offloading schemes typically rely on static rules, fixed thresholds, or centralized controllers. However, such strategies fail to adapt effectively in dynamic environments like disaster zones, where the network topology changes rapidly, tasks arrive unpredictably, and resources are constrained. Moreover, UAVs have limited flight time and battery capacity, requiring intelligent energy-aware strategies for both navigation and task processing. These complexities demand a learning-based approach that can dynamically optimize task offloading decisions based on current environmental and system conditions.

Reinforcement Learning (RL) presents a compelling solution to this problem. As a model-free learning technique, RL enables autonomous agents to learn optimal policies by interacting with the environment and receiving feedback in the form of rewards or penalties [11]. By formulating the task offloading problem as a Markov Decision Process (MDP), UAVs can learn to make decisions that minimize energy consumption and latency while maximizing system performance and reliability [[12], [13], [14]].

This research proposes a Reinforcement Learning-based Task Offloading Framework for UAV-assisted edge-enabled WSNs [15,16], specifically designed for disaster monitoring applications. The proposed system leverages UAVs not only for data collection but also for real-time task processing. By using PPO, a robust and stable policy gradient method, the system dynamically adapts to network changes, improving latency, energy efficiency, and overall reliability in mission-critical disaster environments.

The major contributions of this paper are as follows. (1) A novel framework, ETORL-UAV, is proposed by integrating PPO to enable intelligent, energy-aware, and delay-sensitive task offloading in UAV-assisted edge-enabled WSNs for disaster monitoring. (2) A dynamic MDP based formulation is presented, incorporating key parameters such as residual energy, task urgency, link reliability, and UAV constraints for optimal offloading policy learning.

### Related work

UAVs have become crucial for energy-efficient task execution and communication in disaster monitoring WSNs. M. Dhuheir et al. [17] proposed a meta-RL framework for UAV-based Wireless Power Transfer (WPT) and data collection, outperforming traditional algorithms in energy efficiency and adaptability. Hao Sun et al. [18] introduced a g-MAPPO-based strategy optimizing UAV flight paths and energy fairness in WRSNs . A. Goel et al. [19] developed a hybrid backscatter-based model for joint charging and data collection using convex optimization. Hassaan Hydher et al. [20] proposed a hierarchical UAV deployment strategy using matching and search algorithms to reduce cost and complexity. A. K. Gupta and M. R. Bhatnagar [21] explored game-theoretic UAV charging with pricing strategies based on Nash equilibrium. R. Wang et al. optimized UAV trajectories and recharge scheduling to reduce mission completion time. H. Sheng et al. presented DEHRA-WO, a whale optimization-based dual energy harvesting routing protocol, improving energy use and minimizing packet loss. The above existing methodologies often exhibit key limitations, such as reliance on static heuristics, insufficient adaptability to dynamic and unpredictable disaster environments, and inadequate joint optimization of energy consumption, latency, and task urgency. Many prior works focus on isolated aspects like UAV trajectory or charging optimization without a holistic approach to real-time task offloading under constrained resources. Furthermore, limited integration of intelligent decision-making mechanisms restricts responsiveness in mission-critical scenarios. These gaps underscore the need for an adaptive, energy-aware, and learning-driven framework, motivating the design of the proposed ETORL-UAV system based on PPO for efficient task offloading in edge-enabled WSNs.

## Method details

### Network architecture

**Fig. 2:**
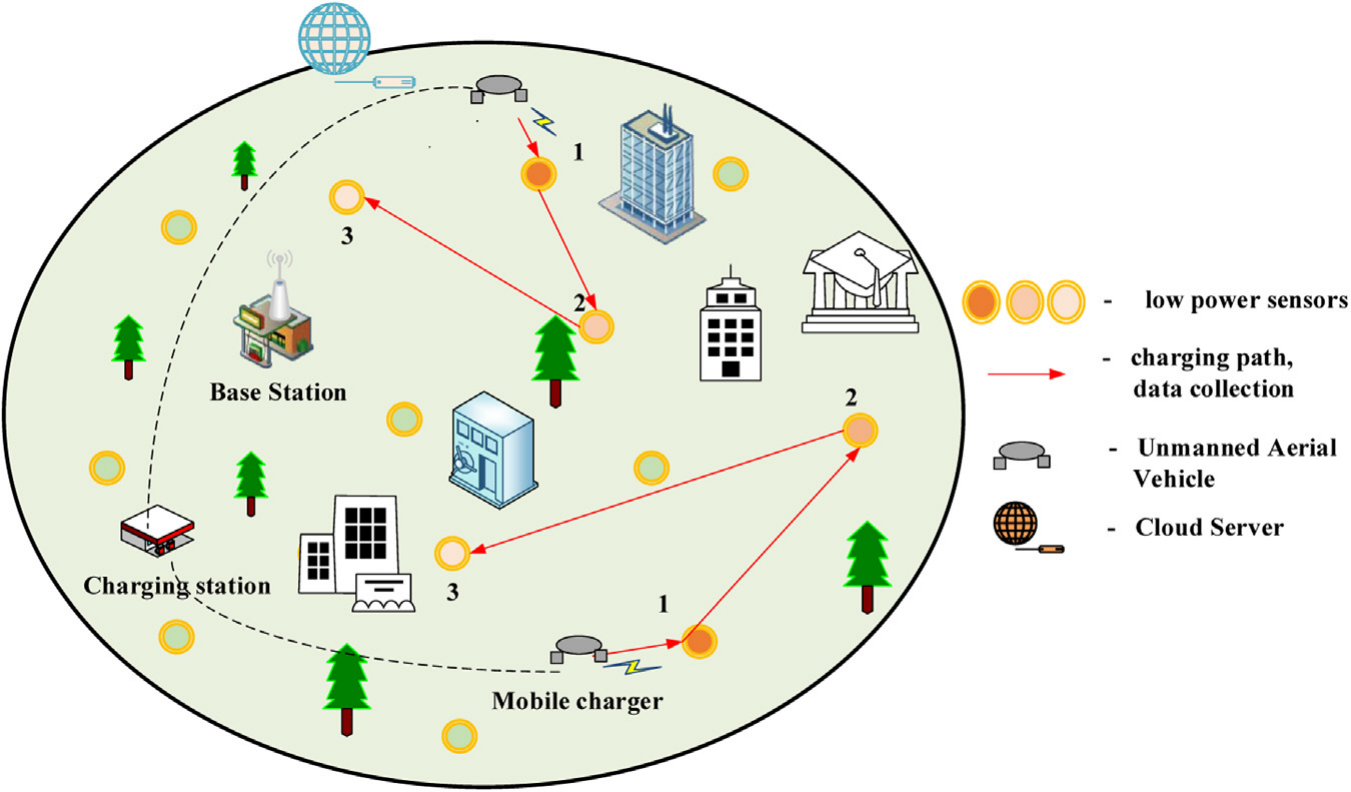
Proposed Network Architecture over disaster prone environment.

The proposed system targets a post-disaster environment where WSNs are deployed for situational monitoring shown in Fig. 2. The architecture is composed of three key components:

(1) $$\mathrm{Sensor}\;\mathrm{Nodes}\left(\mathrm{SNs}\right):\text{Denoted}\;\text{as}\;S=\left\{s_{1},s_{2},\ldots ,s_{N}\right\}$$

these nodes are dispersed across the disaster-struck region and are responsible for sensing environmental parameters such as temperature, humidity, or structural stress. Each node is constrained in energy, processing capability, and communication range.

(2) $$\mathrm{UAVs}:\text{Represented}\;\text{by}\;U=\left\{u_{1},u_{2},\ldots ,u_{M}\right\},$$

UAVs serve as mobile edge computing units. They hover or fly over the WSN, collect data from SNs, execute offloaded tasks, and occasionally relay information to the cloud.

**Cloud Server**: A remote processing unit that handles overflow tasks or tasks that require high computational resources beyond the capabilities of UAVs. Each Sensor S_i_ generates computational tasks T_i_ that can either be processed locally, offloaded to a UAV U_j_, or forwarded to the cloud server.

### Task and energy model

Each task T_i_ is characterized by two primary parameters:

**Data Size** D_i_ (in bits): the amount of data to be transmitted and processed and **Computation Requirement** C_i_ (in CPU cycles): the computational intensity of the task.

If the task is processed locally on the Si, the energy consumed is:

(3) $$E_{i}^{local}=k.f_{i}^{2}.C_{i}$$

where, k is the effective switched capacitance depending on the hardware, C_i_ is the CPU frequency of the sensor node.

For offloading to UAV uju_j, the energy required for transmission is:

(4) $$E_{i,j}^{Comm}=P_{t}\frac{D_{i}}{R_{i,j}}$$

where, $P_{t}$ is the transmit power of the sensor node, $R_{i,j}$ is the achievable data rate, given by the Shannon formula

(5) $$R_{i,j}=B.log_{2}(1+\left(\frac{P_{t}h_{i,j}}{N_{0}}\right)$$

where, $h_{i,j}$is the channel gain between S_i_ and u_j_, $N_{0}$ is the noise power spectral density.

This model ensures the task allocation mechanism evaluates energy constraints and link quality before deciding where to process each task. Such modeling is critical in disaster scenarios where energy conservation directly impacts network longevity and response efficiency.

### UAV mobility and energy model

UAVs are battery-powered and follow a pre-defined trajectory or adaptively move to serve tasks. Their energy model includes two parts, the flying energy part is,

(6) $$E_{j}^{fly}=P_{f}.t_{j},$$

where, $P_{f}\;$is the UAVs propulsion power,$t_{j}$ is the duration of the UAV's flight.

(7) $$\text{The}\;\text{computational}\;\text{energy},\;\;E_{j}^{comp}=K_{u}.f_{j}^{2}C_{i},$$

where, $K_{u}$ is the UAVs switched capacitance, f_j_ is the UAV's processing frequency.

These UAVs are energy-constrained and need to optimize their path and computing load to ensure full coverage and task handling without premature battery depletion. The mobility-aware energy model allows the system to incorporate aerial dynamics into the offloading policy to enhance efficiency and fairness in task allocation.

### Reinforcement learning formulation

We model the decision-making process for task offloading as a **MDP** with the goal of learning an optimal policy that minimizes energy consumption and latency.

### State space

At time t, the state S_t_ captures the environment context

(8) $$s_{t}=\left[E_{i},E_{j},D_{i},C_{i},h_{i,j}\right]$$

where, E_i_, E_j_ are current energy levels of sensor node S_i_ and UAV Uj, D_i_, C_i_ are data size and required computation cycles, h_i,j_ is the channel quality between S_i_ and Uj. This state representation enables the learning agent to make energy and latency aware decisions for task placement in real-time. Capturing both resource availability and wireless channel conditions ensures optimal performance even under unpredictable disaster-area dynamics.

### Action space

(9) $$a_{t}\in \left\{0,1,2\right\}$$

where, 0 is local processing on SN, 1 is Offload to UAV and 2 is Offload to Cloud

This discrete action space simplifies the decision-making while capturing all possible processing options available in the network.

### Reward function

The reward function is designed to minimize a weighted sum of energy consumption and latency

(10) $$r_{t}=-\left(\alpha \cdot E_{total}+\beta \cdot L_{total}\right)$$

where, E_total_ is the total energy consumed, L_total_ is the total latency incurred (transmission + computation) and α, β are the balancing weights for energy and delay. By carefully shaping the reward function, the agent can learn policies that prolong network lifetime and ensure time-sensitive data is processed promptly in disaster scenarios. Table 1 gives the components used in learning algorithm.

**Table 1:** Metrics defined under learning algorithm.

MDP Component	Definition
State (sₜ)	[Eᵢ, Eⱼ, Dᵢ, Cᵢ, hᵢⱼ], -Energy levels, task properties, and channel conditions
Action (aₜ)	{0 = Local Processing, 1 = Offload to UAV, 2 = Offload to Cloud}
Reward (rₜ)	−(α⋅E_total_+β⋅L_total_) -used to penalize high energy and latency
Policy Update	Clipped surrogate objective using PPO

### Learning algorithm

We employ PPO**,** a stable policy-gradient method. The objective is to maximize:

(11) $$max_{\theta }E[min(r_{t}\left(\theta \right).A_{t},clip\left(r_{t}\left(\theta \right),1-\in ,1+\in \right).A_{t}$$

(12) $$r_{t}\left(\theta \right)=\frac{\pi_{\theta }\left(a_{t}|s_{t}\right)\;}{{\pi_{\theta }}_{old}\left(a_{t}|s_{t}\right)}$$

Eq. (12) be the probability ratio between new and old policies, A_t_ be the advantage function estimating the relative value of action $a_{t},$ϵ be the clip range to ensure stable updates

This RL-based offloading strategy enables UAVs to learn optimal decisions in dynamic disaster scenarios, achieving energy-efficient and low-latency operations. This clipped surrogate objective helps in constraining the policy updates to a trust region, thereby improving training robustness and convergence speed.

**Fig. 3:**
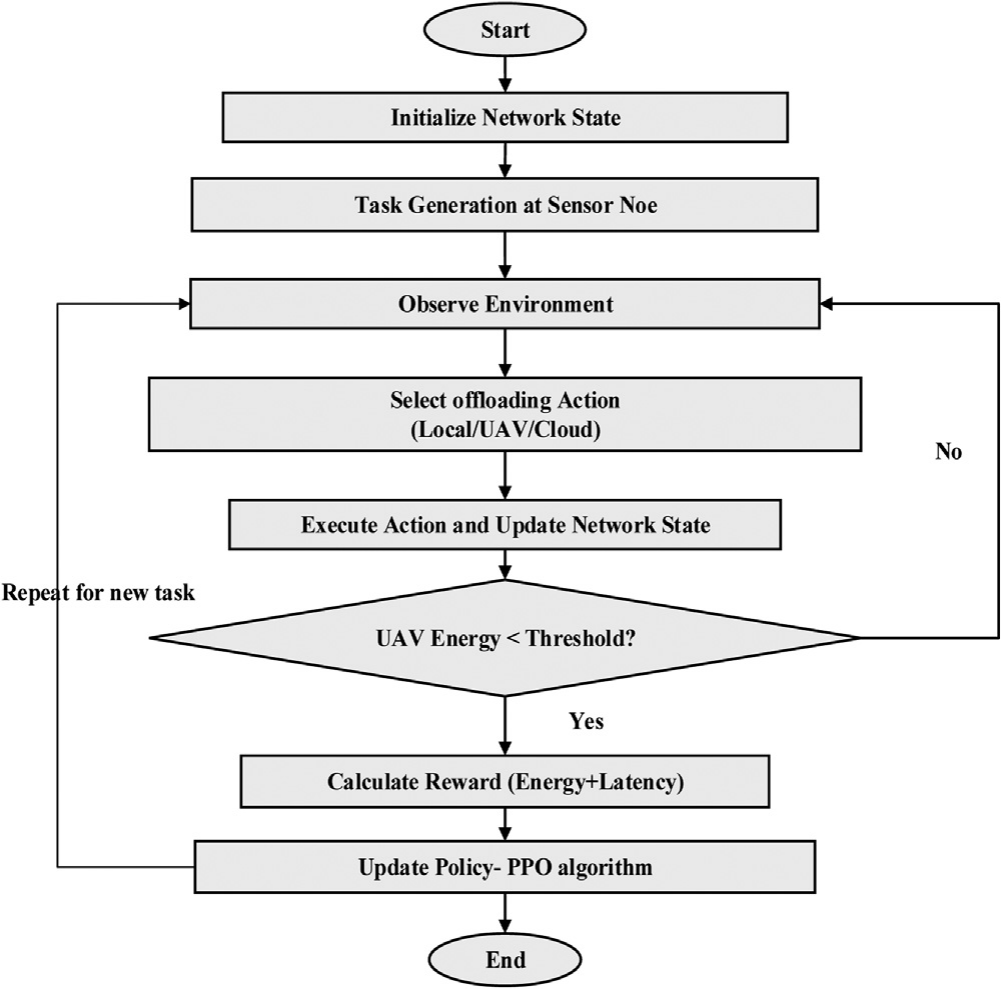
Flow chart for the proposed ETORL-UAV framework.

In the proposed ETORL-UAV framework, we design an intelligent task offloading strategy to enhance energy efficiency and reduce computational latency in UAV-assisted WSNs [22] deployed in remote or disaster-struck environments. The methodology shown in Fig. 3 begins with a decentralized architecture where sensor nodes are responsible for environmental data collection and have the ability to offload computational tasks. Given the limited energy and processing capacity of these nodes, the role UAVs becomes pivotal as mobile edge servers that dynamically collect data and offer computational assistance. Each sensor node periodically generates a task characterized by its data volume and the number of computational cycles required. Based on real-time system conditions such as energy levels of the UAVs and sensor nodes, task size, network latency, and channel quality, the offloading decision is made. The sensor node has three options: process the task locally, offload it to the nearby UAV, or send it to a remote cloud server when edge resources are either unavailable or overloaded.

To learn the optimal task offloading policy, the environment is modeled as MDP, capturing the dynamics of task generation, wireless communication conditions, energy consumption, and mobility of UAVs. We adopt PPO, a stable and efficient reinforcement learning algorithm, to guide the decision-making agent. The agent observes the state of the system, which includes energy levels, distances, task demands, and channel quality, and selects the best action from the defined action space. The reward mechanism of the PPO agent is carefully crafted to penalize high energy consumption and excessive latency, thereby encouraging policies that minimize both. UAVs follow predefined yet adaptable trajectories to interact with sensor nodes, update their policies on the fly using collected experience, and adapt to changing environmental and network dynamics. During training, the PPO agent iteratively updates its policy to maximize the cumulative reward, effectively balancing local processing and offloading strategies. Over time, the model converges to an optimal offloading strategy that ensures task execution with minimal energy usage and latency. In this cooperative learning environment, UAVs not only serve as mobile computing infrastructure but also dynamically adjust their routes and service priorities based on the learned policies.

## Method validation

The simulation environment models a 1000 × 1000 m² disaster-affected area where 150 sensor nodes are randomly deployed to monitor the environment. Three UAVs are employed to patrol the region, collect data, and offer edge computing services for task processing. Sensor nodes generate tasks with varying data sizes and computational requirements. Additionally, a high-capacity cloud server is available for offloading, albeit with higher latency compared to UAV-based edge processing. Table 2 gives the values over the parameters used for simulation analysis.

**Table 2:** Simulation parameters for ETORL-UAV.

Parameter	Value
Number of Sensor Nodes (N)	150
Number of UAVs (M)	3
Simulation Area	1000 × 1000 m²
Bandwidth (B)	1 MHz
Sensor Node Transmission Power	0.1 W
UAV Propulsion Power (P_f)	50 W
UAV Flight Speed	20 m/s
Training Episodes	2000

**Fig. 4:**
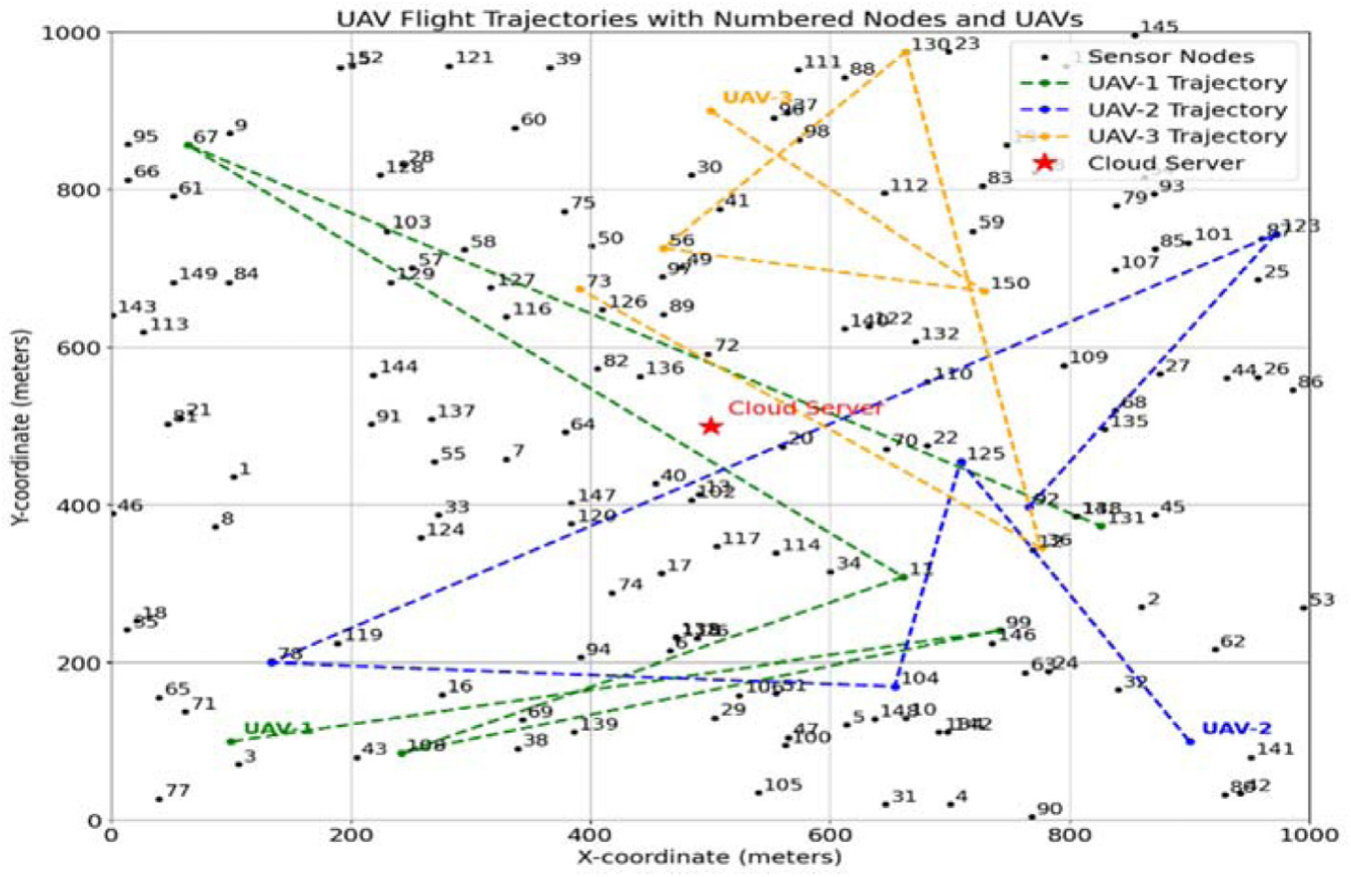
Path Taken by UAVs to reach Sensor Node.

**Fig. 5:**
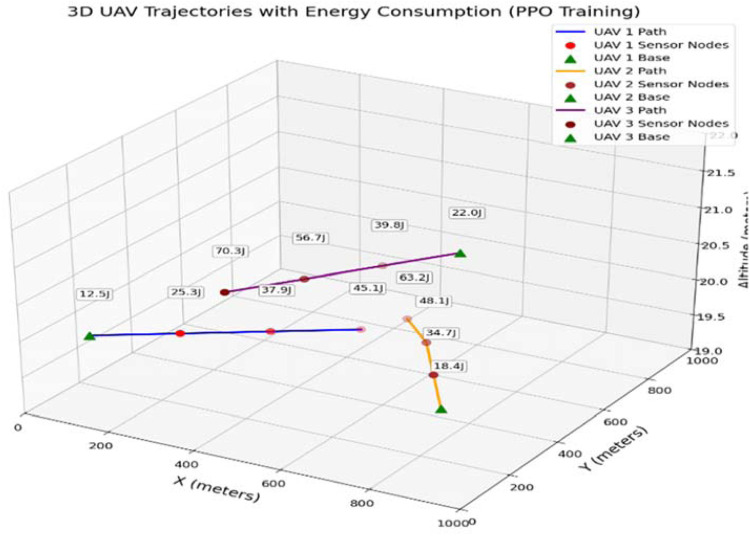
Trajectories of UAVs according to energy level of Nodes.

The simulation of the ETORL-UAV framework was implemented in Python using libraries such as NumPy, Matplotlib, and Stable-Baselines3. A custom Gym environment was developed to model the MDP involving UAVs and sensor nodes in a 1000 × 1000 m² disaster-affected area. PPO was employed to learn optimal task offloading decisions based on factors like energy level, task size, and latency. Each sensor node generates tasks, and UAVs act as mobile edge servers to process or offload them efficiently. The simulation evaluates performance based on energy consumption, task latency, and network lifetime, highlighting the adaptive efficiency of ETORL-UAV. The two-dimensional (2D) UAV trajectory visualization confirmed the smart routing behavior of ETORL-UAV as shown in Fig. 4. UAVs initiated from designated charging stations and navigated through optimal paths to the sensor nodes with critical tasks, minimizing both the distance traveled and energy consumed. These UAVs only accessed the cloud server when on-board resources were inadequate, highlighting an efficient offloading decision mechanism. The path planning ensured that high-priority nodes were serviced early, while also balancing energy reserves and minimizing overlaps between multiple UAVs. Additionally, a three-dimensional (3D) UAV trajectory graph was used to demonstrate how ETORL-UAV manages UAV movement in realistic spatial domains as shown in Fig. 5. Each UAV’s path is annotated with energy consumption values, showcasing the economical use of onboard energy resources. The altitude remained steady at approximately 20 m, which is ideal for line-of-sight communication and effective WPT. These 3D plots reinforced the system’s ability to perform intelligent altitude-aware navigation while maintaining energy efficiency and coverage.

**Fig. 6:**
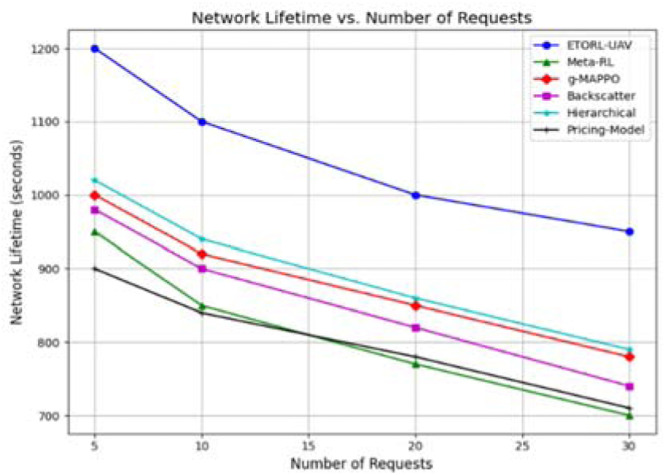
Lifetime comparison with other methods.

To evaluate the performance of the proposed ETORL-UAV framework, a comprehensive simulation study was conducted, comparing it against five recent benchmark algorithms: Meta-RL, g-MAPPO, Backscatter Optimization, Hierarchical Optimization, and Pricing-Model using Game Theory. The comparative analysis was carried out across several performance metrics, including network lifetime, task offloading success rate, energy consumption, and UAV trajectory efficiency. Each algorithm was subjected to different task request loads (5, 10, 20, and 30), and the performance under these dynamic scenarios was recorded and analyzed. The network lifetime analysis revealed that ETORL-UAV consistently delivers superior performance in prolonging the operational time of the WSN as shown in Fig. 6. Across all task loads, ETORL-UAV maintained a network lifetime exceeding 950 s, even under high request density (30 requests). In comparison, the other approaches particularly the Pricing Model and Meta-RL experienced more rapid declines, falling below 750 s at higher task volumes. This prolonged lifetime of the ETORL-UAV strategy is primarily attributed to its reinforcement learning-enabled energy-aware decision-making and efficient UAV scheduling, which prevents redundant movements and minimizes idle energy waste.

**Fig. 7:**
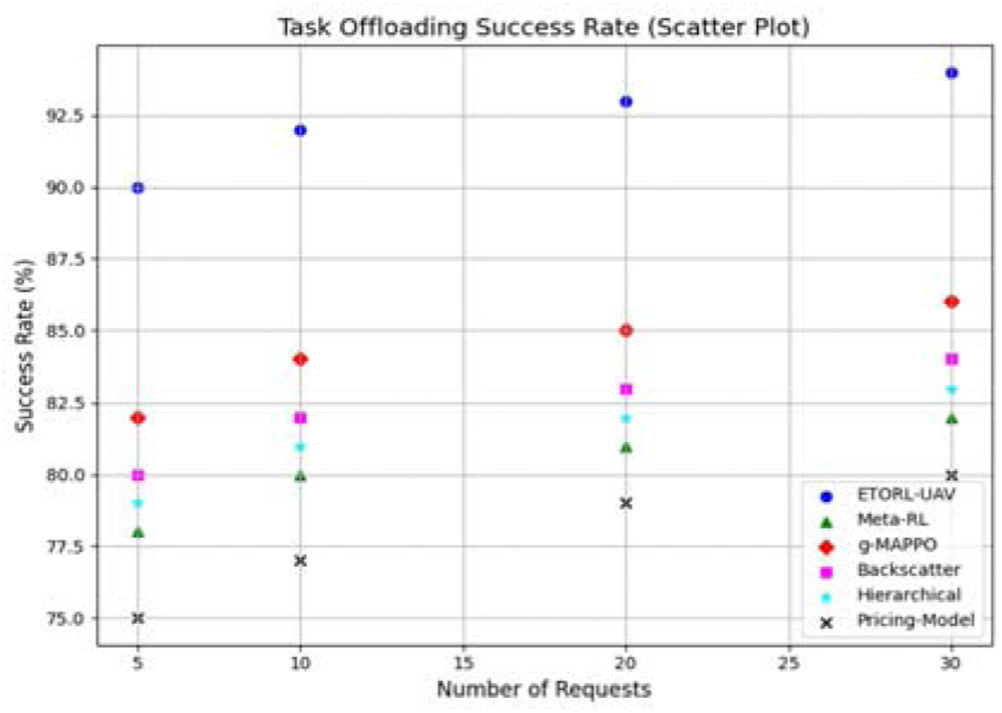
Task offloading Performance comparison.

**Fig. 8:**
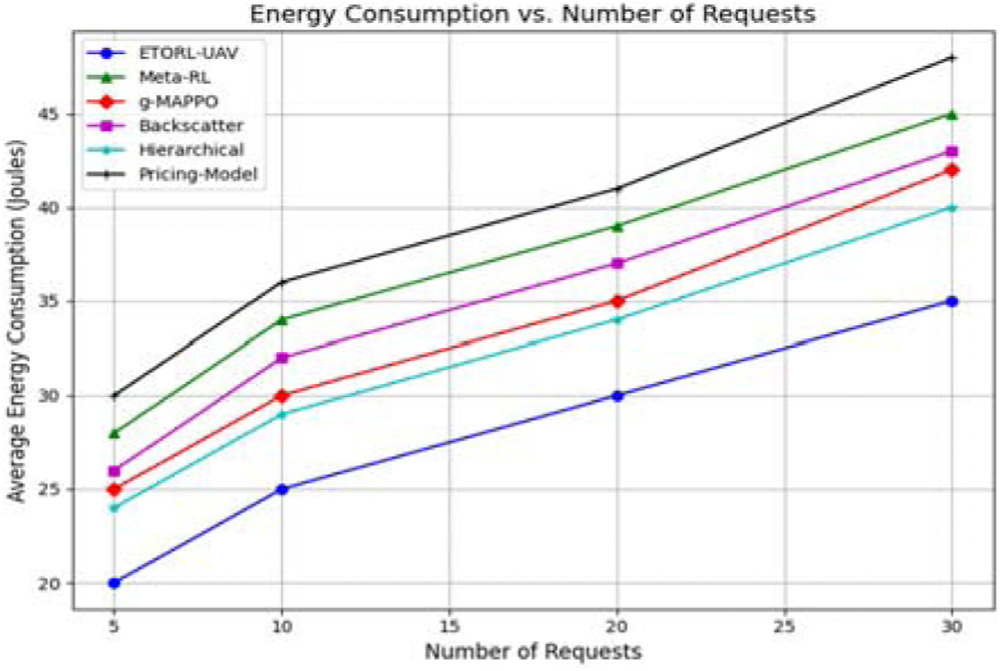
Energy Usage comparison with other methods.

The task offloading success rate was examined through a scatter plot, illustrating the percentage of successful task completions as the number of requests increased given in Fig. 7. ETORL-UAV demonstrated a consistently high success rate, maintaining above 93 % across all scenarios. This high efficiency is the result of intelligent task selection, energy-threshold-aware offloading, and dynamic routing. In contrast, methods like Meta-RL and g-MAPPO achieved only around 82–85 %, while the Pricing Model dropped significantly lower. The superior success rate of ETORL-UAV emphasizes its capability to handle heavy and distributed workloads effectively in edge-enabled WSNs, a critical requirement for disaster and real-time monitoring. The energy consumption analysis provided further evidence of ETORL-UAV’s efficiency. The proposed method consumed significantly less average energy compared to the other approaches, particularly at higher task densities shown in Fig. 8. At 30 requests, ETORL-UAV consumed only around 35 Joules, whereas the Pricing Model required over 47 Joules. This reduction is directly linked to optimized trajectory planning, minimal task overlap, and reinforcement learning-based energy control strategies that dynamically adjust offloading and charging operations based on the UAV’s energy state and task urgency.

Overall, the simulation results strongly validate the effectiveness of the proposed ETORL-UAV framework in achieving energy-efficient, reliable, and timely task execution in UAV-assisted WSNs. It consistently surpasses existing benchmark methods across multiple performance dimensions. The reinforcement learning-driven task offloading and routing logic, combined with energy-aware UAV behavior, makes ETORL-UAV a robust and scalable solution for disaster response, environmental monitoring, and other critical IoT-driven applications.

## Limitations

The proposed ETORL-UAV framework assumes moderately stable environments, which may not capture extreme real-world dynamics like rapid topology changes or complex flight physics. Communication channels are modeled ideally, without considering severe fading or obstructions

## Ethics statements

Not Applicable

## CRediT authorship contribution statement

**C.N. Vanitha:** Conceptualization, Methodology, Project administration, Supervision, Writing – original draft. **P. Anusuya:** Software, Data curation, Visualization, Writing – original draft. **Rajesh Kumar Dhanaraj:** Formal analysis, Validation, Resources, Writing – review & editing. **Dragan Pamucar:** Investigation, Methodology, Supervision, Writing – review & editing. **Mahmoud Ahmad Al-Khasawneh:** Funding acquisition, Validation, Writing – review & editing.

## Declaration of competing interest

The authors declare that they have no known competing financial interests or personal relationships that could have appeared to influence the work reported in this paper.

## Data Availability

Data will be made available on request.
